# Impact of modified‐release opioid use on clinical outcomes following total hip and knee arthroplasty: a propensity score‐matched cohort study

**DOI:** 10.1111/anae.16070

**Published:** 2023-06-26

**Authors:** S. Liu, A. E. Patanwala, J. M. Naylor, N. Levy, R. Knaggs, J. A. Stevens, B. Bugeja, D. Begley, K. E. Khor, E. Lau, R. Allen, S. Adie, J. Penm

**Affiliations:** ^1^ Faculty of Medicine and Health, School of Pharmacy The University of Sydney Sydney NSW Australia; ^2^ Department of Pharmacy Prince of Wales Hospital Randwick NWS Australia; ^3^ Faculty of Medicine and Health, School of Pharmacy The University of Sydney Sydney NSW Australia; ^4^ Pharmacy Department Royal Prince Alfred Hospital Camperdown NSW Australia; ^5^ Orthopaedic Department, Whitlam Orthopaedic Research Centre Liverpool Hospital Liverpool NSW Australia; ^6^ South Western Sydney Clinical School University of New South Wales Sydney NSW Australia; ^7^ Department of Anaesthesia and Peri‐operative Medicine West Suffolk Hospital Bury St. Edmunds UK; ^8^ School of Pharmacy University of Nottingham and Primary Integrated Community Services Nottingham UK; ^9^ School of Clinical Medicine, St Vincent's Clinical Campus University of New South Wales Sydney NSW Australia; ^10^ School of Medicine University of Notre Dame Sydney NSW Australia; ^11^ Department of Pain Management Prince of Wales Hospital Sydney NSW Australia; ^12^ Department of Pain Management Prince of Wales Hospital Sydney NSW Australia; ^13^ Department of Pain Management Prince of Wales Hospital Sydney NSW Australia; ^14^ Prince of Wales Clinical School University of New South Wales Medicine and Health Sydney NSW Australia; ^15^ Department of Pharmacy St George Hospital Kogarah NSW Australia; ^16^ Pain Management Unit St George Hospital Kogarah NSW Australia; ^17^ St George and Sutherland Clinical School University of New South Wales Sydney NSW Australia; ^18^ Faculty of Medicine and Health, School of Pharmacy The University of Sydney Sydney NSW Australia

**Keywords:** adverse events, modified‐release, opioids, total hip arthroplasty, total knee arthroplasty

## Abstract

Modified‐release opioids are often prescribed for the management of moderate to severe acute pain following total hip and knee arthroplasty, despite recommendations against their use due to increasing concerns regarding harm. The primary objective of this multicentre study was to examine the impact of modified‐release opioid use on the incidence of opioid‐related adverse events compared with immediate‐release opioid use, among adult inpatients following total hip or knee arthroplasty. Data for total hip and knee arthroplasty inpatients receiving an opioid analgesic for postoperative analgesia during hospitalisation were collected from electronic medical records of three tertiary metropolitan hospitals in Australia. The primary outcome was the incidence of opioid‐related adverse events during hospital admission. Patients who received modified with or without immediate‐release opioids were matched to those receiving immediate‐release opioids only (1:1) using nearest neighbour propensity score matching with patient and clinical characteristics as covariates. This included total opioid dose received. In the matched cohorts, patients given modified‐release opioids (n = 347) experienced a higher incidence of opioid‐related adverse events overall, compared with those given immediate‐release opioids only (20.5%, 71/347 vs. 12.7%, 44/347; difference in proportions 7.8% [95%CI 2.3–13.3%]). Modified‐release opioid use was associated with an increased risk of harm when used for acute pain during hospitalisation after total hip or knee arthroplasty.

## Introduction

Total hip or knee arthroplasty are common worldwide [1]. Each require varied pain management approaches to cover the acute postoperative period that routinely include opioid analgesics for the management of moderate to severe acute pain [2]. A recent study of 86,058 patients reported that over 80% of patients used opioids for the management of acute pain following total hip or knee arthroplasty [2]. The types of opioid formulations prescribed for post‐surgical pain after these procedures may include immediate‐release and modified‐release formulations. Modified‐release opioid formulations were introduced in the late 1990s to provide prolonged, stable serum opioid concentrations [3] and have since become widely used for the management of post‐surgical pain [4]. A cohort study highlighted that modified‐release opioids accounted for over 30% of all opioids prescribed after surgery, and patients undergoing total hip or knee arthroplasty were over 10 times more likely to be prescribed a modified‐release opioid [4]. This increase has been driven by the suggestion that modified‐release opioids may provide improved pain relief, reduce the need for frequent immediate‐release opioid administration [5], and may be less addictive due to fewer peak and trough plasma concentrations [6]. These suggestions were further potentiated by aggressive marketing and sponsorship of the ‘*Pain as the 5th Vital Sign*’ campaign by pharmaceutical companies [6]. A study by Reuben et al. published in 1999 reporting superior outcomes associated with modified‐release opioid use after anterior cruciate ligament surgery was cited 116 times before it was retracted for falsification of data [7]. The impact of this study on the literature and practice before its retraction is unknown. A randomised controlled trial of 59 patients admitted for inpatient rehabilitation following total knee arthroplasty reported that modified‐release oxycodone use was associated with improved physical function and a shorter length of hospital stay compared with placebo [8]. As a result of these findings and other studies [9, 10], modified‐release opioid formulations were recommended for use in some postoperative analgesia guidelines [11] and enhanced recovery pathways [12], which are increasingly being used after total hip and knee arthroplasty procedures [13].

However, several studies showing positive outcomes associated with modified‐release opioids used inappropriate comparators such as placebo [8], epidurals [9], used lower oral morphine equivalent doses [10] or were not conducted in acute postoperative settings [8]. Collectively, data describing the impact of modified‐release opioid use on clinical outcomes appear to be mixed, with several studies showing no benefits of modified‐release compared with immediate‐release opioid use [14, 15, 16]. Recent concerns have been raised regarding the challenges of titrating modified‐release opioid doses to rapidly changing pain intensities during the postoperative period, which may lead to supratherapeutic serum opioid concentrations, sedation and opioid‐induced ventilatory impairment [17]. It has been suggested that some modified‐release opioids may have an 8‐h duration of effect despite 12‐hourly dosing [18], which may contribute towards breakthrough pain, higher opioid consumption [16] and a higher incidence of opioid‐related adverse events [4, 16]. Initiation of modified‐release opioid formulations is also associated with an increased risk of long‐term opioid use compared with those given immediate‐release opioids only [19]. These concerns have led to recommendations against the use of modified‐release opioids for acute pain by numerous regulatory bodies including the UK Royal College of Anaesthetists and its Faculty of Pain Medicine [20], the United States Centers for Disease Control and Prevention [21] and the American Pain Society [22] and the Australian and New Zealand College of Anaesthetists and its Faculty of Pain Medicine [17]. Furthermore, the product licence of modified‐release oxycodone in Australia [23], the UK [24] and the USA [25] specifically advises against the use of modified‐release oxycodone within the first 24 h after surgery, first 12–24 h after surgery and immediate postoperative periods, respectively.

Despite these concerns and recommendations, modified‐release opioids are still commonly used for the management of acute pain following total hip and knee arthroplasty [4]. There is a need to compare modified‐release and immediate‐release opioid use following these procedures, and to determine whether the use of modified‐release opioids is safe or harmful. The primary objective of this study was to examine the impact of modified‐release opioid use on the incidence of opioid‐related adverse drug events compared with immediate‐release opioid use among adult hospital inpatients following primary total hip or knee arthroplasty. Secondary objectives included examining the impact of modified‐release opioid use compared with immediate‐release opioid use on length of hospital stay and 28‐day readmission rate following primary total hip or knee arthroplasty.

## Methods

This study was approved by the South Eastern Sydney Local Health District Human Research Ethics Committee. We conducted a multicentre retrospective cohort study in three hospitals in New South Wales, Australia, a 450‐bed tertiary teaching hospital (Hospital A), a 650‐bed tertiary teaching hospital (Hospital B) and a 375‐bed teaching hospital (Hospital C). Electronic medical record data for people admitted between 1 January 2018 and 31 December 2021 were reviewed.

Patients aged > 18 y who underwent a primary unilateral or bilateral total hip or knee arthroplasty as defined by the Australian Classification of Health Intervention 10th Edition procedure codes [26] (online Supporting Information Table S1) and received an opioid analgesic after surgery during hospital stay were included. Exclusion criteria included the use of opioid formulations for oncology or opioid substitution therapy (for opioid dependence) purposes, same‐day surgery patients and repeat admissions.

Patients were divided into two groups: a modified‐release opioid group, which comprised all eligible patients who were administered any modified‐release opioid analgesic formulation and, optionally, any immediate‐release opioid formulations during admission; and an immediate‐release opioid only group, which comprised all eligible patients who were administered any immediate‐release opioid analgesic formulation and no modified‐release opioid formulations during admission. Propensity score matching was used to adjust for known confounders and is described below.

De‐identified demographic data were collected from the electronic medical records (Millennium Powerchart, Cerner Corporation, North Kansas City, MO, USA). The International Classification of Diseases Australian Modification (ICD‐10‐AM) diagnosis codes as assigned by hospital clinical coders were used to identify comorbidities and opioid‐related adverse events [27]. The onset of ICD‐10‐AM conditions of interest relative to hospital admission were determined using the Australian Institute of Healthcare and Welfare Onset Flags [28]. We calculated the Charlson comorbidity index using ICD‐10‐AM codes and respective weightings used in previous literature where a higher total score indicated a greater disease burden [29]. Opioid‐related adverse events were captured using ICD‐10‐AM codes adapted from previous research [30] and author consensus. Opioid‐related adverse events were defined as the incidence of one or more of the following: constipation; nausea and vomiting; heartburn; diarrhoea; abdominal pain; somnolence; delirium (not due to infective sources or substance withdrawal); dizziness; headache; hallucinations; sleep disturbances; respiratory depression; urinary retention; pruritis; dry mouth; opioid toxicity; in‐hospital fall; and muscle spasm (online Supporting Information Table S2). The incidence of opioid‐related adverse events in total was defined as the incidence of at least one of the aforementioned opioid‐related adverse events during hospital stay. Length of hospital stay was calculated by subtracting the admission date from the discharge date. The 28‐day readmission rate was calculated using the proportion of discharged patients who are readmitted within 28 days of hospital discharge.

Medication administration data during hospital stay were extracted from medication administration records, including administration of: opioid analgesics; paracetamol; non‐steroidal anti‐inflammatory drugs; gabapentinoids (gabapentin and pregabalin); benzodiazepines; and laxatives. Opioid administration was defined as the documented administration of: buprenorphine (sublingual 200 mcg strength, injection and transdermal formulations); codeine; fentanyl; hydromorphone; morphine; methadone; oxycodone (including oxycodone/naloxone); tapentadol; or tramadol. We excluded formulations used for antidiarrhoeal, antitussive and opioid substitution purposes (including liquid methadone, sublingual buprenorphine (400 mcg, 2 mg and 8 mg strengths) and injectable buprenorphine) from analysis. Oral morphine milligram‐equivalent dosages were calculated using established conversion factors [31]. Modified‐release opioid formulations were defined as any opioid formulation (including oral and transdermal preparations) with a slow release rate of the active ingredient [17].

The primary outcome of this study was the incidence of at least one opioid‐related adverse event. Secondary outcomes included length of hospital stay and 28‐day readmission rate. Based on previous literature [4] and clinical estimates, we assumed the proportion of patients who experience opioid‐related adverse events were 20% in the modified‐release opioid group and 10% in the immediate‐release opioid only group. Using an α of 0.05 in a two‐sided test and 80% power, a total of 199 patients were required in each group. Power analyses were conducted using G*Power software (version 3.1.9.6). Univariate analyses were conducted to compare baseline characteristics between patients in the modified‐release and immediate‐release opioid groups. Categorical variables were compared using Fisher's exact test. Continuous non‐normally distributed variables were examined using the Mann–Whitney U‐test.

Propensity score matching was used to reduce the effects of confounding and obtain comparable groups [32]. The probability of receiving a modified‐release vs. an immediate‐release opioid was calculated using a multivariable logistic regression model [32]. Covariates in the propensity score model were identified using existing literature [33] and author consensus. Covariates included: average opioid dose received per day in hospital (oral morphine milligram equivalent dose received during total admission divided by hospital length of stay); age; sex; Charlson comorbidity index; type of surgery (total hip or knee arthroplasty); all extracted comorbidities; alcohol; opioid; tobacco and substance use disorders; homelessness; pre‐operative opioid use (including pre‐operative modified‐release or immediate‐release opioid use); concurrent paracetamol, non‐steroidal anti‐inflammatory drug, laxative, benzodiazepine or gabapentinoid administration; and opioids given during inpatient stay.

Patients who received modified‐release opioids were matched to those who received immediate‐release opioids only using nearest‐neighbour matching, with a 1:1 matching ratio without replacement [32]. The calliper width was calculated by using 0.1 times the standard deviation of the logit of the propensity score, as described in previous literature [34]. If a patient in the immediate‐release opioid group was not found for a given modified‐release opioid group patient, that patient was excluded. The balance of covariates between the matched groups were assessed using absolute standardised mean differences (SMD) rather than hypothesis testing as SMDs are not affected by sample size [35]. A SMD value < 0.1 indicated negligible differences between groups [35].

After propensity score matching, binary outcomes (incidence of opioid‐related adverse events and 28‐day readmission rate) between the modified‐release and immediate‐release opioid groups were compared using Fisher's exact test. The 95%CI for the difference in proportions between groups for the primary outcome was reported. Length of hospital stay was compared between groups using the Mann–Whitney U‐test.

A p value < 0.05 was considered statistically significant. All statistical analyses were performed using SPSS Statistics (version 27.0, IBM Corp, Armonk, NY, USA) and R (version 3.1.2, The R Foundation, Vienna, Austria).

## Results

**Figure 1:**
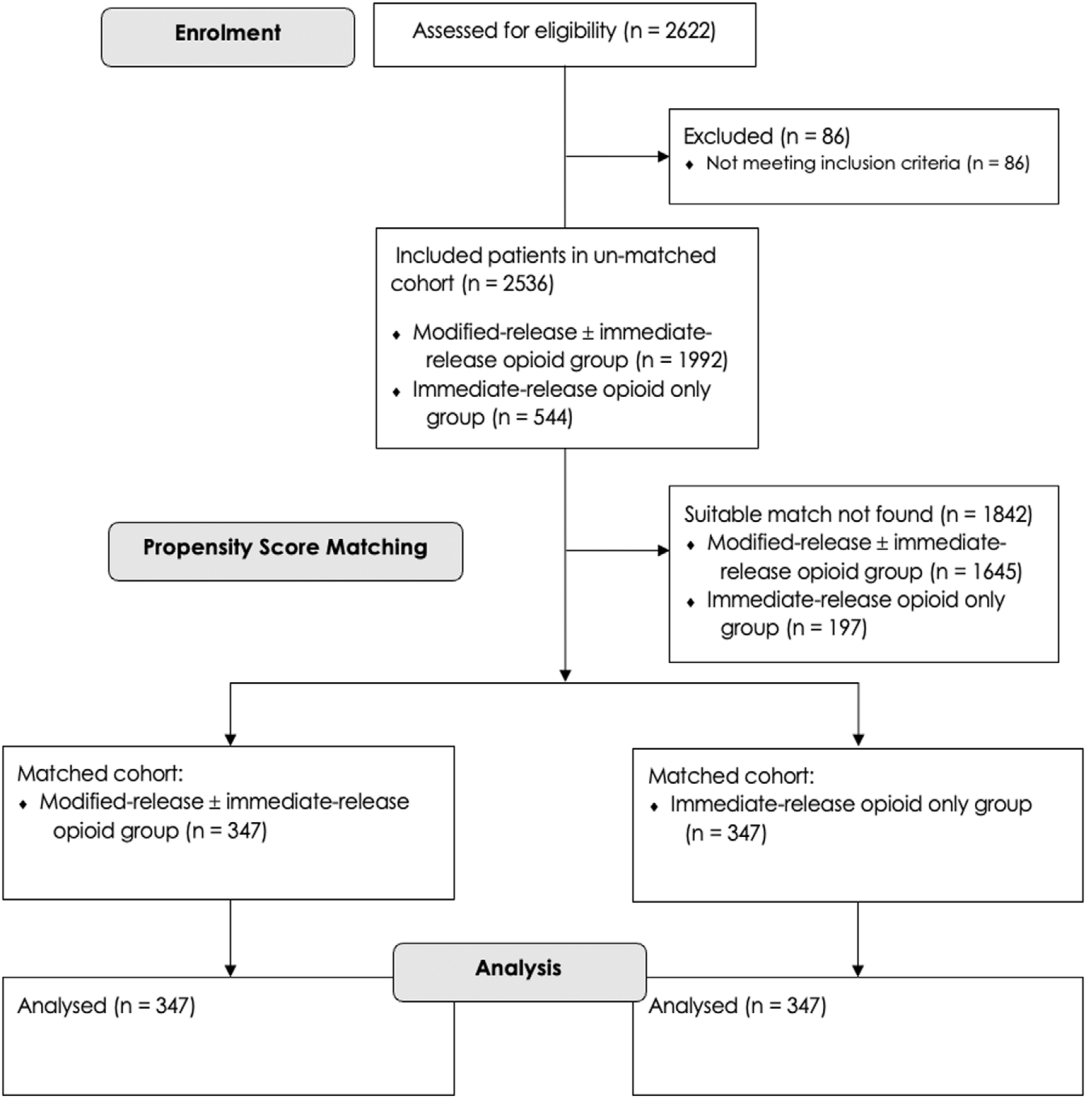
Patient flow diagram.

During the study period, 2622 patients underwent total hip or knee arthroplasty, of which 2536 met the study inclusion criteria. Of these, there were 1992 patients in the modified‐release opioid group (78.5%) and 544 patients in the immediate‐release opioid only group (21.5%) in the unmatched sample. Within the matched study population, there were 347 patients in each group. A match within the calliper limits for the remaining patients were not found. A patient flow diagram is shown in Fig. 1.

Among the unmatched cohorts, baseline and clinical characteristics differed (Table 1). The mean (SD) age of the modified‐release opioid cohort was 68.8 (12.1) y and 68.4 (12.8) y in the immediate‐release opioid only cohort. The median (range) Charlson comorbidity index score was 0 (0–9) in the modified‐release opioid cohort and 0 (0–8) in the immediate‐release opioid only cohort. During inpatient stay, patients given modified‐release opioids were more likely to be co‐administered gabapentinoids (14.2%, 282/1992 vs. 10.1%, 55/544, SMD 0.12) and laxatives (82.2%, 1637/1992 vs. 77.2%, 420/544, SMD 0.13) than patients given immediate‐release opioids only (Table 2). The main modified‐release opioids given included modified‐release oxycodone with naloxone (71.2%, 1419/1992) and modified‐release tapentadol (23.1%, 461/1992). Among the modified‐release opioid group and the immediate‐release opioid only group, the main immediate‐release opioids given included immediate‐release oxycodone (86.6%, 1726/1992 vs. 87.5%, 476/544) and immediate‐release tapentadol. Patients given modified‐release opioids received a greater median daily oral morphine milligram equivalent dose compared with those given immediate‐release opioids only (33.3 [1.0–296.3] vs. 11.7 [0.1–285.7], SMD 0.68; Table 2).

**Table 1:** Baseline characteristics of patients undergoing total hip or knee arthroplasty before and after propensity score matching for the modified‐ and immediate‐release opioid or the immediate‐release opioid only groups. Values are mean (SD), number (proportion) or median (IQR [range]).

	Before propensity score matching			After propensity score matching		
	MR ± IR opioids	IR opioids only	SMD	MR ± IR opioids	IR opioids only	SMD
	n = 1992	n = 544		n = 347	n = 347	
Age; y	68.8 (12.1)	68.4 (12.8)	0.04	69.3 (11.7)	69.4 (12.1)	0.01
Sex; male	912 (45.8%)	222 (40.8%)	0.10	144 (41.5%)	144 (41.5%)	0.00
Facility						
Hospital A	811 (40.7%)	349 (64.2%)	1.14	189 (54.5%)	186 (53.6%)	0.00
Hospital B	135 (6.8%)	29 (5.3%)	1.95	26 (7.5%)	25 (7.2%)	0.00
Hospital C	1046 (52.5%)	166 (30.5%)	0.44	132 (38%)	136 (39.2%)	0.02
Charlson comorbidity index score	0 (0–1 [0–9])	0 (0–1 [0–8])	0.00	0 (0–1 [0–8])	0 (0–1 [0–8])	0.00
0	1444 (72.5%)	383 (70.4%)	0.04	250 (72%)	242 (69.7%)	0.04
1	255 (12.8%)	78 (14.3%)	0.03	39 (11.2%)	51 (14.7%)	0.09
2	240 (12%)	70 (12.9%)	0.03	47 (13.5%)	48 (13.8%)	0.00
3–5	37 (1.9%)	11 (2%)	0.00	11 (3.2%)	5 (1.4%)	0.09
>6	15 (0.8%)	2 (0.4%)	0.12	2 (0.6%)	1 (0.3%)	0.09
Comorbidities						
Heart failure	27 (1.4%)	5 (0.9%)	0.00	7 (2%)	4 (1.2%)	0.08
Hypertension	159 (8%)	35 (6.4%)	0.08	25 (7.2%)	29 (8.4%)	0.04
Atrial fibrillation	72 (3.6%)	14 (2.6%)	0.06	13 (3.7%)	10 (2.9%)	0.06
Acute myocardial infarction	4 (0.2%)	0	0.00	4 (1.2%)	0	0.09
Atherosclerotic heart disease	5 (0.3%)	2 (0.4%)	0.00	2 (0.6%)	0	0.09
Pulmonary heart disease	5 (0.3%)	1 (0.2%)	0.00	1 (0.3%)	1 (0.3%)	0.00
Acute renal failure	46 (2.3%)	13 (2.4%)	0.00	10 (2.9%)	9 (2.6%)	0.00
Chronic kidney disease	35 (1.8%)	18 (3.3%)	0.07	9 (2.6%)	11 (3.2%)	0.00
Type 2 diabetes mellitus	385 (19.3%)	116 (21.3%)	0.05	72 (20.7%)	75 (21.6%)	0.02
Liver disease	2 (0.1%)	4 (0.7%)	0.20	1 (0.3%)	1 (0.3%)	0.00
Chronic obstructive pulmonary disease	26 (1.3%)	7 (1.3%)	0.00	4 (1.2%)	6 (1.7%)	0.08
Sleep apnoea	33 (1.7%)	10 (1.8%)	0.00	5 (1.4%)	6 (1.7%)	0.08
Neuropathic pain	1 (0.1%)	0	0.00	1 (0.3%)	0	0.00
Chronic pain	49 (2.5%)	5 (0.9%)	0.07	3 (0.9%)	3 (0.9%)	0.00
History of shock	1 (0.1%)	0	0.00	1 (0.3%)	0	0.00
Obesity	33 (1.7%)	6 (1.1%)	0.08	2 (0.6%)	4 (1.2%)	0.00
Constipation	220 (11%)	44 (8.1%)	0.10	26 (7.5%)	29 (8.4%)	0.04
Ulcerative colitis	2 (0.1%)	3 (0.6%)	0.23	2 (0.6%)	3 (0.9%)	0.00
Inflammatory bowel disease	1 (0.1%)	0	0.00	1 (0.3%)	0	0.00
Diverticulitis	3 (0.2%)	0	0.00	3 (0.9%)	0	0.09
Osteoarthritis	1647 (82.7%)	433 (79.6%)	0.08	277 (79.8%)	275 (79.3%)	0.02
Anxiety	21 (1.1%)	6 (1.1%)	0.00	4 (1.2%)	4 (1.2%)	0.00
Depression	4 (0.2%)	4 (0.7%)	0.18	1 (0.3%)	1 (0.3%)	0.00
Dementia	23 (1.2%)	3 (0.6%)	0.00	1 (0.3%)	3 (0.9%)	0.09
History of delirium	28 (1.4%)	7 (1.3%)	0.00	6 (1.7%)	3 (0.9%)	0.09
Bipolar disorder	0	0	N/A	0	0	N/A
Schizophrenia	3 (0.2%)	1 (0.2%)	0.00	0	1 (0.3%)	0.00
Alcohol use disorder	25 (1.3%)	6 (1.1%)	0.00	6 (1.7%)	4 (1.2%)	0.08
Opioid use disorder	6 (0.3%)	5 (0.9%)	0.15	1 (0.3%)	2 (0.6%)	0.00
Tobacco use disorder	694 (34.8%)	190 (34.9%)	0.00	121 (34.9%)	121 (34.9%)	0.00
Substance use disorder	3 (0.2%)	3 (0.6%)	0.20	0	1 (0.3%)	0.00
Homelessness	1 (0.1%)	0	0.00	1 (0.3%)	0	0.00
ACHI Procedures						
Primary total knee arthroplasty	1032 (51.8%)	244 (44.9%)	0.14	171 (49.3%)	163 (47%)	0.04
Primary total hip arthroplasty	960 (48.2%)	300 (55.1%)	0.14	176 (50.7%)	184 (53%)	0.04

MR, modified‐release; IR, immediate‐release; SMD, absolute standardised mean difference; N/A, not applicable; ACHI, Australian Classification of Health Interventions.

A history of gastro‐intestinal haemorrhage, irritable bowel syndrome and pneumonia were considered as comorbidities, but are not displayed in the table as none of the included patients had these comorbidities.

**Table 2:** Medications administered to patients undergoing total hip or knee arthroplasty before and after propensity score matching for the modified‐ and immediate‐release opioid or the immediate‐release opioid only groups. Values are number (proportion) or median (IQR [range]).

	Before propensity score matching			After propensity score matching		
	MR ± IR opioids	IR opioids only	SMD	MR ± IR opioids	IR opioids only	SMD
	n = 1992	n = 544		n = 347	n = 347	
Total pre‐operative opioid use	1067 (53.6%)	224 (41.2%)	0.26	121 (34.9%)	122 (35.2%)	0.00
Pre‐operative MR opioid use	836 (42%)	25 (4.6%)	0.78	24 (6.9%)	24 (6.9%)	0.00
Pre‐operative IR opioid use	952 (47.8%)	219 (40.3%)	0.16	119 (34.3%)	117 (33.7%)	0.00
Inpatient medication use						
Opioid analgesics, daily MME	33.3 (19.3–53.2 [1.0–296.3])	11.7 (6.0–21.3 [0.1–285.7])	0.68	18.8 (11.1–30.6 [0.1–296.3])	14.0 (7.5–26.3 [0.1–285.7])	0.03
MR opioid use	1992 (100%)	N/A	N/A	347 (100%)	N/A	N/A
Buprenorphine	35 (1.8%)	N/A	0.17	6 (1.7%)	N/A	N/A
Fentanyl	9 (0.5%)	N/A	0.00	1 (0.3%)	N/A	N/A
Tramadol	104 (5.2%)	N/A	0.25	23 (6.6%)	N/A	N/A
Morphine	11 (0.6%)	N/A	0.15	2 (0.6%)	N/A	N/A
Tapentadol	461 (23.1%)	N/A	0.60	62 (17.9%)	N/A	N/A
Oxycodone (total)	1503 (75.5%)	N/A	1.53	274 (79%)	N/A	N/A
Oxycodone alone	104 (5.2%)	N/A	0.25	14 (4%)	N/A	N/A
Oxycodone with naloxone	1419 (71.2%)	N/A	1.43	261 (75.2%)	N/A	N/A
IR opioid use	1849 (92.8%)	544 (100%)	0.30	332 (95.7%)	347 (100%)	N/A
Buprenorphine	1 (0.1%)	1 (0.2%)	0.00	0	0	N/A
Codeine	23 (1.2%)	14 (2.6%)	0.17	5 (1.4%)	8 (2.3%)	0.07
Fentanyl	160 (8%)	44 (8.1%)	0.00	32 (9.2%)	31 (8.9%)	0.00
Hydromorphone	32 (1.6%)	20 (3.7%)	0.14	10 (2.9%)	10 (2.9%)	0.00
Tramadol	117 (5.9%)	29 (5.3%)	0.04	23 (6.6%)	22 (6.3%)	0.04
Morphine	258 (13%)	44 (8.1%)	0.15	40 (11.5%)	31 (8.9%)	0.09
Methadone	8 (0.4%)	10 (1.8%)	0.24	3 (0.9%)	5 (1.4%)	0.00
Tapentadol	276 (13.9%)	122 (22.4%)	0.22	57 (16.4%)	53 (15.3%)	0.03
Oxycodone	1726 (86.6%)	476 (87.5%)	0.00	316 (91.1%)	307 (88.5%)	0.09
Paracetamol	1984 (99.6%)	543 (99.8%)	0.00	346 (99.7%)	346 (99.7%)	0.00
NSAIDs	1017 (51.1%)	253 (46.5%)	0.08	167 (48.1%)	164 (47.3%)	0.02
COX‐2 inhibitors	575 (28.9%)	145 (26.7%)	0.04	93 (26.8%)	94 (27.1%)	0.00
Benzodiazepines	168 (8.4%)	44 (8.1%)	0.00	25 (7.2%)	31 (8.9%)	0.07
Gabapentinoids	282 (14.2%)	55 (10.1%)	0.12	44 (12.7%)	37 (10.7%)	0.06
Total laxative use	1637 (82.2%)	420 (77.2%)	0.13	278 (80.1%)	275 (79.3%)	0.02
Softener laxatives	22 (1.1%)	2 (0.4%)	0.10	4 (1.2%)	2 (0.6%)	0.00
Bulk‐forming laxatives	1595 (80.1%)	388 (71.3%)	0.22	254 (73.2%)	256 (73.8%)	0.02
Osmotic laxatives	1607 (80.7%)	409 (75.2%)	0.15	271 (78.1%)	268 (77.2%)	0.02
Stimulant laxatives	1797 (90.2%)	463 (85.1%)	0.16	303 (87.3%)	302 (87%)	0.00

MR, modified‐release; IR, immediate‐release; SMD, absolute standardised mean difference; MME, oral morphine milligram equivalents; N/A, not applicable; NSAIDs, non‐steroidal anti‐inflammatory drugs; COX‐2, cyclo‐oxygenase‐2.

After propensity score matching, the absolute SMDs were < 0.1, indicating similar baseline characteristics between the groups and an adequate match (Table 1; online Supporting Information Figure S1). The mean (SD) age of the modified‐release opioid group was 69.3 (11.7) y and 69.4 (12.1) y for the immediate‐release opioid only group. The median Charlson comorbidity index score of both groups was 0 (0–8). Approximately 80% of patients in both groups were administered laxatives during their inpatient stay (278/347 in the modified‐release opioid group vs. 275/347 in the immediate‐release opioid only group, SMD 0.02, Table 2). Co‐administration of benzodiazepines was identified in 7.2% (25/347) of patients in the modified‐release opioid group and 8.9% (31/347) of patients in the immediate‐release opioid only group. The main modified‐release opioids given included modified‐release oxycodone with naloxone (75.2%, 261/347) and modified‐release tapentadol (17.9%, 62/347). Among the matched modified‐release opioid group and immediate‐release opioid only group, the main immediate‐release opioids given included immediate‐release oxycodone (91.1%, 316/347 vs. 88.5%, 307/347) and immediate‐release tapentadol (16.4%, 57/347 vs. 15.3%, 53/347). After matching, the median daily opioid dose was similar between modified‐release and immediate‐release opioid groups (18.8 [0.1–296.3] vs. 14.0 [0.1–285.7], SMD 0.03, Table 2).

In the matched cohorts, the incidence of opioid‐related adverse events overall was greater among patients given modified‐release opioids compared with immediate‐release opioids only (20.5%, 71/347 vs. 12.7%, 44/347, p = 0.008; 95%CI for difference in proportions 2.3–13.3%). A greater proportion of patients receiving modified‐release opioids experienced constipation (5.8%, 20/347 vs. 1.7%, 6/347, p = 0.008) and in‐hospital falls (1.7%, 6/347 vs. 0%, 0/347, p = 0.031) compared with those receiving immediate‐release opioids only. The median (IQR [range]) length of hospital stay was longer for patients given modified‐release opioids compared with immediate‐release opioids alone (8 (4–11 [1–121]) vs. 5 (3–9 [1–90]) days, p < 0.001). Rates of hospital readmission within 28 days were similar between groups (3.7%, 13/347 vs. 4.3%, 15/347, p = 0.847) (Table 3).

**Table 3:** Opioid‐related adverse drug events (ORADEs) in the modified‐ and immediate‐release opioid group or the immediate‐release opioid only matched groups. Values are number (proportion).

Outcome	MR ± IR opioids	IR opioids only	p value
	n = 347	n = 347	
Total all ORADEs	71 (20.5%)	44 (12.7%)	0.008
Total gastro‐intestinal ORADEs	37 (10.7%)	15 (4.3%)	0.002
Constipation	20 (5.8%)	6 (1.7%)	0.008
Nausea and vomiting	16 (4.6%)	8 (2.3%)	0.144
Heartburn	1 (0.3%)	1 (0.3%)	1.000
Diarrhoea	0	0	N/A
Abdominal pain	1 (0.3%)	0	1.000
Other gastro‐intestinal ORADEs	0	1 (0.3%)	1.000
Total central nervous system ORADEs	13 (3.7%)	8 (2.3%)	0.376
Somnolence	3 (0.9%)	0	0.249
Delirium^a^	7 (2%)	6 (1.7%)	1.000
Dizziness	2 (0.6%)	2 (0.6%)	1.000
Headache	1 (0.3%)	1 (0.3%)	1.000
Hallucinations	0	0	N/A
Sleep disturbance	1 (0.3%)	0	1.000
Other central nervous system ORADEs	0	0	N/A
Respiratory depression	9 (2.6%)	8 (2.3%)	1.000
Urinary retention	14 (4%)	15 (4.3%)	1.000
Other ORADEs (total)	7 (2.0%)	3 (0.9%)	0.340
Pruritis	1 (0.3%)	2 (0.6%)	1.000
Dry mouth	1 (0.3%)	0	1.000
Opioid toxicity	0	0	N/A
In‐hospital fall	6 (1.7%)	0	0.031
Muscle spasm	0	1 (0.3%)	1.000

N/A, not applicable.

^a^ Not due to infective sources or substance withdrawal.

## Discussion

In this multicentre propensity score matched cohort study, modified‐release opioid use in the acute postoperative period is associated with an increased risk of experiencing opioid‐related adverse events after total hip or knee arthroplasty compared with those given immediate‐release opioids only. Further, patients given modified‐release opioids experienced a higher incidence of constipation and in‐hospital falls, as well as a longer length of hospital stay compared with patients given immediate‐release opioids only. Our findings build on a growing body of evidence highlighting the harms associated with modified‐release opioid use for acute surgical pain. For instance, a study conducted in 2021 showed that modified‐release opioid use was associated with an increased incidence of opioid‐related adverse events and longer length of hospital stay among a broader population of surgical hospital inpatients [4]. Similarly, a double‐blind randomised controlled trial of 200 patients undergoing total hip or knee arthroplasty reported the addition of modified‐release morphine to usual care for acute postoperative pain led to more adverse effects such as vomiting and oversedation, yet minimal improvements in pain intensity, compared with patients who did not receive modified‐release opioids [15].

These findings have important implications for the healthcare system and associated costs. The present study showed patients given modified‐release opioids had a longer length of hospital stay by an additional 3 days. Data from the Australian Institute of Health and Welfare suggest that each additional day of acute inpatient care costs up to AU$1500 (£785, €909) per patient receiving total hip or knee arthroplasty [36]. Assuming 60% of patients undergoing total hip or knee arthroplasty are given modified‐release opioids after surgery and over 100,000 total hip or knee arthroplasty procedures are performed in Australia annually [37], based on prolonged length of hospital stay alone, current modified‐release opioid prescribing practices may be associated with additional healthcare costs of over AU$250 million per year in Australia. Further studies are warranted to identify whether modified‐release opioids may be beneficial in some patient cohorts, such as patients with certain comorbidities necessitating the need for modified‐release opioid use.

Oxycodone with naloxone accounted for more than two‐thirds of modified‐release opioid formulations prescribed in the study cohort. The addition of naloxone, a mu‐opioid receptor antagonist which acts locally on the gastro intestinal tract, to oxycodone was promoted and used to reduce the effect of opioid‐induced constipation, one of the most common adverse events associated with opioid use [38]. Despite this, constipation was found to be more common among patients given modified‐release opioids after total hip or knee arthroplasty compared with those given immediate‐release opioids only in this study. Existing literature as summarised by a 2013 meta‐analysis of randomised controlled trials suggest that the use of naloxone either alone or in combination with oxycodone is superior to placebo for the management of opioid‐induced constipation [39]. However, all included trials reporting on the efficacy of naloxone were conducted in the context of chronic non‐malignant pain rather than acute pain [39]. Evidence from two randomised controlled trials included in a 2017 literature review assessing the impact of naloxone on opioid‐induced constipation among patients with post‐surgical pain showed no difference in rates of constipation between groups [40]. In post‐surgical settings, laxatives are often prescribed for prophylaxis of constipation. In the present study, approximately 80% of the study cohort was prescribed a laxative after surgery. Therefore, our study findings reinforce the results of previous research suggesting that the addition of naloxone has a limited role in the context of acute pain. The extensive use of oxycodone with naloxone after total hip and knee arthroplasty may thus represent a source of unnecessary healthcare utilisation. Future strategies to reduce the use of oxycodone with naloxone for acute pain may provide an opportunity to optimise the use of healthcare resources whilst reducing harms associated with modified‐release opioid use.

Some guidelines continue to recommend the use of modified‐release opioids for the management of pain after total hip or knee arthroplasty [11, 12]. The modified‐release opioid prescribing practices observed in the present study arose due to the enhanced recovery pathways used in the included study hospitals. These advise the provision of modified‐release opioids alongside immediate‐release opioid and non‐opioid analgesia after total hip and knee arthroplasty to facilitate faster functional recovery and a shorter length of hospital stay. Other authors have identified additional reasons for the initiation of modified‐release opioids following total hip and knee arthroplasty such as reduced nursing workload due to less frequent analgesic needs, particularly in regional or rural settings in which nurse‐to‐patient staffing ratios may be lower than urban settings [5]. However, there are increasing concerns that the slow onset and offset of modified‐release opioid formulations may prevent appropriate dose titration to fluctuating acute pain requirements [17], which may contribute towards inadequate pain relief [16] and an increased risk of opioid‐related adverse events including opioid‐induced ventilatory impairment [17]. The pharmacokinetic profile of modified‐release oxycodone, the main opioid formulation administered in the present cohort, has been shown to provide inadequate analgesia over the conventional 12‐h dosing period, which may lead to breakthrough pain, higher opioid consumption and subsequent dose‐related harm [18]. Previous literature suggest an 8‐h dosing frequency of modified‐release oxycodone may be more appropriate [18].

Furthermore, the use of modified‐release opioids has been associated with longer term harm such as persistent opioid use, which in turn increases the risk of opioid tolerance, dependence and even mortality [17]. These risks are heightened by co‐administration of opioids with other sedating drugs including benzodiazepines, which were prescribed to 8% of the study cohort against practice guidelines [17]. Thus, consideration of patient safety‐related to opioid use in the context of co‐administered therapies is advised. All patients commenced on modified‐release opioid formulations for acute pain should have a clear opioid tapering and cessation plan to reduce the risk of persistent opioid use and associated harms. This recommendation is supported by several professional, regulatory and advisory bodies globally [17, 20, 21, 22]. In light of the conclusions drawn from this study and previous research highlighting the increased harms and length of hospital stay associated with modified‐release opioid use, re‐evaluation of guidelines and hospital policies to remove recommendations for modified‐release opioid use after surgery may improve the alignment of clinical practice to international guidelines.

A strength of this study is the use of data from multiple hospital sites, which improves the generalisability of our findings. The collection of data on medications given to patients rather than prescription data provides a more accurate representation of patients' medication exposure during inpatient stay. Patients' collected baseline characteristics were balanced using propensity score matched analysis to reduce bias associated with confounding while achieving external validity by using observational data. However, there were several limitations to this study. First, due to the retrospective nature of the study, data quality may be limited by the accuracy of the electronic medical record data used and causality could not be conclusively established. Data on medications administered through patient‐controlled and regional analgesia routes, or within operating theatres, intensive care units and high dependency care contexts were not available for collection, which may result in the under‐representation of medications administered. The ICD‐10‐AM data used to capture comorbidities and opioid‐related adverse events are limited by coding accuracy and are not opioid‐specific; thus, the incidence of comorbidities and opioid‐related adverse events may be underestimated and/or miscategorised. For example, only a small proportion of the study cohort was categorised with chronic pain as a comorbidity, yet a significant proportion had pre‐operative opioid use. Also, the ICD‐10‐AM codes for respiratory depression may not capture all relevant centrally‐impaired breathing disorders that encompass opioid‐induced ventilatory impairment. As data on the specific day on which each opioid‐related adverse event occurred, a temporal relationship between modified‐release or immediate‐release opioid use and the incidence of opioid‐related adverse events could not be established. Despite the use of propensity score matching to minimise bias, there may have been unmeasured or uncollected variables affecting patients' likelihood of receiving modified‐release opioids during hospital stay, such as individual patient or prescriber preferences. Patients' opioid doses were matched between the modified‐release and immediate‐release opioid‐only groups. However, there remained a higher opioid dose among the modified‐release opioid group compared with the immediate‐release opioid‐only group following matching (below the pre‐specified threshold for significance), which may be a confounder to the increase in opioid‐related adverse events identified in the modified‐release opioid group. In patient pain scores and functional outcomes were not collected, thus their impact on modified‐release opioid prescribing is unknown.

After patients receiving modified‐release opioids were matched to those taking immediate‐release opioids only after total hip or knee arthroplasty, the findings of this study suggest that modified‐release with or without immediate‐release opioid use is associated with a higher incidence of opioid‐related adverse events compared with immediate‐release opioid use alone. Specifically, patients given modified‐release opioids experienced more constipation, in‐hospital falls and prolonged hospital stay compared with those given immediate‐release opioids only. This study adds to the increasing body of evidence that modified‐release opioids do not provide benefits over immediate‐release opioids, and supports the existing advice that modified‐release opioids should be avoided in the peri‐operative period [17, 20, 21, 22]. If modified‐release opioids are prescribed postoperatively, use should be avoided within the first 12–24 h after surgery to comply with the product licences, unless within the context of a registered clinical study.

## Supporting information


**Figure S1.** Love plot of covariate balance before and after propensity score matching for the modified‐ and immediate‐opioid vs. the immediate‐release opioid only groups.

**Table S1.** Australian Classification of Health Intervention 10th Edition procedure codes used to identify patients who underwent primary total hip or knee arthroplasty.

**Table S2.** International Classification of Diseases 10th Edition Australian Modification procedure codes used to identify opioid‐related adverse drug events.
