# Supplementary figures and images for: Impact of modified‐release opioid use on clinical outcomes following total hip and knee arthroplasty: a propensity score‐matched cohort study
Source: Anaesthesia. 2023 Jun 26;78(10):1237–48. doi: 10.1111/anae.16070 (PMC10952779; doi:10.1111/anae.16070)

Covariate Balance

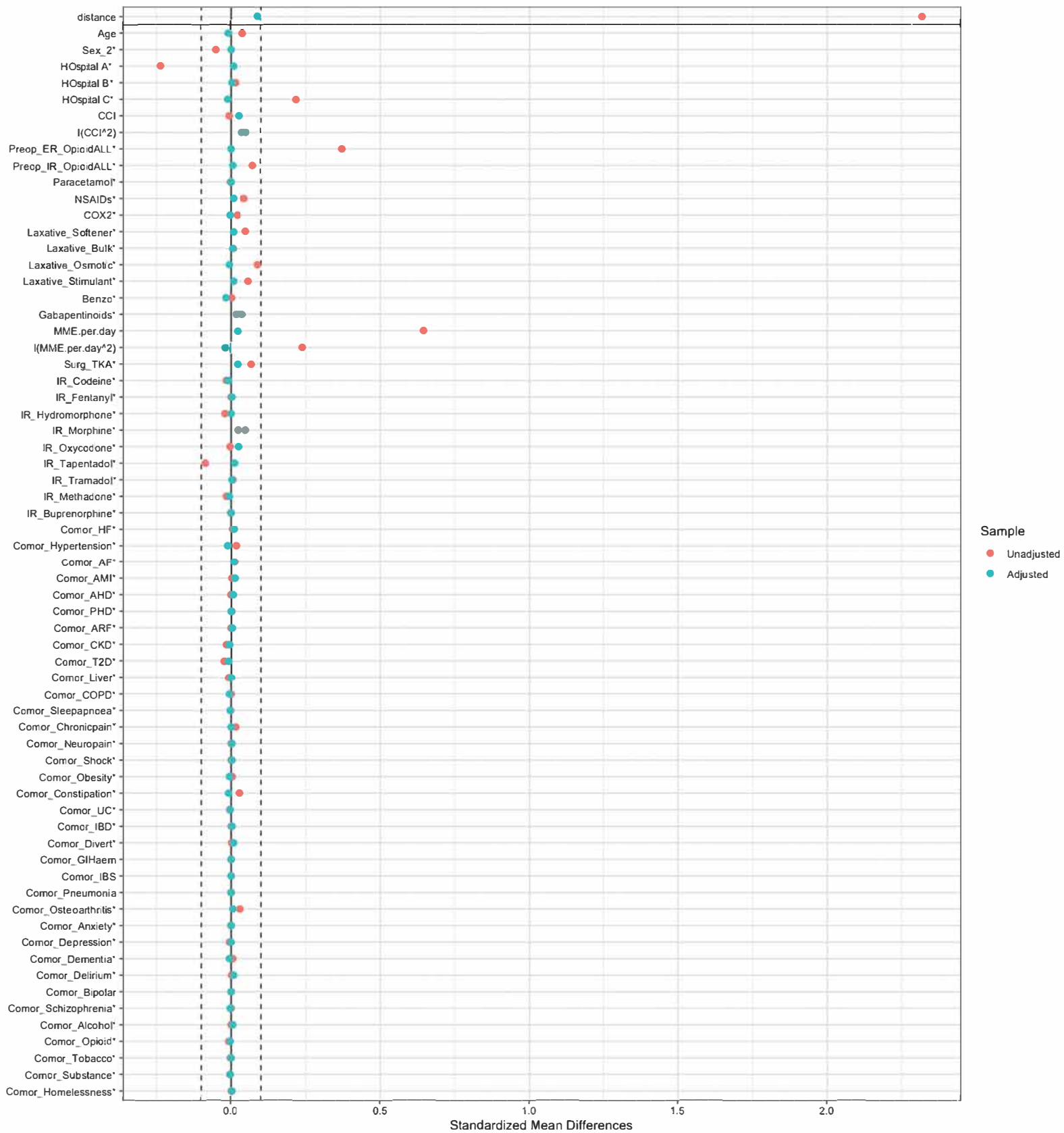

Supplement: Supplementary file 1 — Figure S1. Love plot of covariate balance before and after propensity score matching for the modified‐ and immediate‐opioid vs. the immediate‐release opioid only groups. [file ANAE-78-1237-s003.pdf]
