# Supplementary material for: Impact of modified‐release opioid use on clinical outcomes following total hip and knee arthroplasty: a propensity score‐matched cohort study
Source: Anaesthesia. 2023 Jun 26;78(10):1237–48. doi: 10.1111/anae.16070 (PMC10952779; doi:10.1111/anae.16070)
Supplement: Supplementary file 2 — Table S1. Australian Classification of Health Intervention 10th Edition procedure codes used to identify patients who underwent primary total hip or knee arthroplasty. [file ANAE-78-1237-s002.docx]

**Table S1**. Australian Classification of Health Intervention 10^th^ Edition procedure codes used to identify patients who underwent primary total hip or knee arthroplasty.

| **ACHI procedure code** | **Surgical procedure** |
| --- | --- |
| 49318-00 | Total arthroplasty of hip, unilateral |
| 49319-00 | Total arthroplasty of hip, bilateral |
| 49518-00 | Total arthroplasty of knee, unilateral |
| 49519-00 | Total arthroplasty of knee, bilateral |
| 49521-00 | Total arthroplasty of knee with bone graft to femur, unilateral |
| 49521-02 | Total arthroplasty to knee with bone graft to tibia, unilateral |
| 49524-00 | Total arthroplasty of knee with bone graft to femur and tibia, unilateral |
| 49524-01 | Total arthroplasty of knee with bone graft to femur and tibia, bilateral |

ACHI, Australian Classification of Health Intervention.
