# Supplementary material for: Impact of modified‐release opioid use on clinical outcomes following total hip and knee arthroplasty: a propensity score‐matched cohort study
Source: Anaesthesia. 2023 Jun 26;78(10):1237–48. doi: 10.1111/anae.16070 (PMC10952779; doi:10.1111/anae.16070)
Supplement: Supplementary file 3 — Table S2. International Classification of Diseases 10th Edition Australian Modification procedure codes used to identify opioid‐related adverse drug events. [file ANAE-78-1237-s001.docx]

**Table S2**. International Classification of Diseases 10^th^ Edition Australian Modification procedure codes used to identify opioid-related adverse drug events.

| **Description** | **ICD10-AM Code** | **ICD-10-AM Code Description** |
| --- | --- | --- |
| **Gastrointestinal opioid-related adverse drug events** | | |
| Constipation | K56.7 | Ileus, unspecified |
|  | K56.0 | Paralytic ileus |
|  | K59.0 | Constipation |
| Nausea and vomiting | R11 | Nausea and vomiting |
| Heartburn | R12 | Heartburn |
| Diarrhoea | K52.9 | Diarrhoea, noninfective |
|  | K59.1 | Functional diarrhoea |
| Abdominal pain | R10.4 | Abdominal pain, unspecified |
| Other gastrointestinal adverse event | K91.8 | Other postprocedural disorders of digestive system, not elsewhere classified |
| **Central Nervous System opioid-related adverse drug events** | | |
| Somnolence | R40.0 | Somnolence |
|  | R40.1 | Stupor |
| Delirium | R41.0 | Disorientation, unspecified |
|  | R41.8 | Other and unspecified symptoms and signs involving cognitive functions |
|  | F05.9 | Delirium, unspecified |
| Dizziness | H81.4 | Vertigo of central origin |
|  | R42 | Dizziness and giddiness |
| Headache | R51 | Headache |
| Hallucinations | R44.3 | Hallucinations, unspecified |
| Sleep disturbances | F51.5 | Nightmares |
|  | G47.0 | Insomnia (organic) |
| Other central nervous system adverse event | G97.8 | Other postprocedural disorders of nervous system |
| **Respiratory opioid-related adverse drug events** | | |
| Respiratory depression | J96.0 | Acute respiratory failure |
|  | J95.8 | Other postprocedural respiratory disorders |
|  | J96.9 | Respiratory failure, unspecified |
|  | J95.2 | Acute pulmonary insufficiency following nonthoracic surgery |
|  | R06.8 | Other and unspecified abnormalities of breathing |
|  | R09.0 | Asphyxia |
|  | J80 | Acute respiratory distress syndrome |
|  | J18.2 | Hypostatic pneumonia, unspecified |
|  | R06.0 | Dyspnoea |
| **Genitourinary opioid-related adverse drug events** | | |
| Urinary retention | R33 | Retention of urine |
|  | N99.8 | Other postprocedural disorders of genitourinary system |
|  | R34 | Anuria and oliguria |
| **Other opioid-related adverse drug events** | | |
| Pruritis | L29.9 | Pruritus, unspecified |
| Dry mouth | R68.2 | Dry mouth, unspecified |
| Opioid toxicity | T40.0 | Poisoning by opium |
|  | T40.2 | Poisoning by other opioids |
|  | T40.3 | Poisoning by methadone |
|  | T40.4 | Poisoning by other synthetic narcotics |
|  | T40.6 | Poisoning by other and unspecified narcotics |
| In-hospital fall | W04 | Fall while being carried or supported by other persons |
|  | W06 | Fall involving bed |
|  | W18.0 | Fall from bumping against object |
|  | W18.9 | Unspecified fall on same level |
|  | W18.8 | Other specified fall on same level |
| Muscle spasm | R25.2 | Cramp and spasm |

ICD-10-AM, International Classification of Diseases 10^th^ Edition Australian Modification.
